# Beclin-1 Targeting for Viral Immune Escape

**DOI:** 10.3390/v3071166

**Published:** 2011-07-12

**Authors:** Christian Münz

**Affiliations:** Viral Immunobiology, Institute of Experimental Immunology, University of Zürich, Winterthurerstrasse 190, CH-8057 Zürich, Switzerland; E-Mail: christian.muenz@uzh.ch; Tel.: +41-44-635-3716; Fax: +41-44-635-6883

**Keywords:** autophagy, influenza virus, HIV, HSV, KSHV, immunity, immune evasion

## Abstract

Macroautophagy is a catabolic pathway in eukaryotic cells that has recently been shown to facilitate pathogen detection, pathogen restriction and pathogen-derived antigen presentation to CD4^+^ T cells. Due to these protective functions during immune responses, several pathogens, including RNA and DNA viruses, have developed strategies to inhibit autophagosome generation or maturation. Interestingly, most of the respective viral proteins exert these functions via binding to Beclin-1, an essential macroautophagy protein that constitutes part of the phosphatidylinositol-3 kinase complexes that mark membranes for autophagosome generation and facilitate autophagosome fusion with lyososomes. The viruses that inhibit macroautophagy by this pathway include herpesviruses, HIV and influenza A virus. Inhibition either before or after autophagosome formation seems to benefit their viral replication by different mechanisms, which are discussed here.

## 1. Introduction

Lysosomes and proteasomes are the two main catabolic machineries in eukaryotic cells. Endocytosed, but also autophagocytosed material can reach lysosomes, thereby delivering extra- or intracellular material for degradation, respectively. At least three pathways of autophagy exist for the delivery of cytoplasmic constituents for lysosomal degradation. These are macro-, micro- and chaperone-mediated autophagy [1]. While micro- and chaperone-mediated autophagy occur directly at the lysosomal membrane, macroautophagy can originate at the rough endoplasmic reticulum, Golgi apparatus, outer nuclear or mitochondrial membrane, and the cell membrane [2–10]. During microautophagy a part of the lysosomal membrane invaginates into the lumen of these degradative vesicles and thereby delivers cytosolic substrates for lysosomal hydrolysis. In higher eukaryotes this seems to occur with the help of the chaperone HSC70 [11]. Chaperone-mediated autophagy, however, has been observed in higher eukaryotic cells only after prolonged starvation. Under these conditions, substrates with a signal motif, a permutation of the pentamer KFERQ, get transported across the lysosomal membrane with the help of the lysosome associated membrane protein (LAMP) 2a, cytosolic chaperones and luminal chaperones in the lysosomes. Signal peptide recognition seems to occur via the cytosolic chaperone, and therefore similar substrates are selected for microautophagy and chaperone mediated autophagy in higher eukaryotic cells [11]. Finally, most steady-state turn-over of cytosolic protein aggregates and organelles in lysosomes of higher eukaryotes seems to require macroautophagy for delivering these substrates. Macroautophagy is executed through the interaction of more than 30 autophagy related gene (*atg*) products, whose loss prevents yeast from surviving starvation conditions via this pathway. During this process a double membrane, called a phagophore, engulfs substrates, anchoring the inner autophagosomal membrane to the cargo via Atg8/LC3, which is covalently attached to phosphatidylethanolamine in the autophagosomal membrane via an ubiquitin-like conjugation reaction involving the E1-, E2- and E3-like enzymes Atg7, Atg3 and Atg12-Atg5/Atg16L1 [12,13]. Atg8/LC3 is either directly bound by a cargo protein, like the outer mitochondrial membrane protein NIX for mitophagy [14,15], or by anchor proteins, like p62/sequestosome 1, optineurin, NBR1 or NDP52 [16–20], that cross-link Atg8/LC3 with polyubiquitinated substrates. The completed autophagosome then fuses with late endosomes or directly with lysosomes, whereby the inner membrane and its cargo get degraded by lysosomal hydrolysis. Autophagosome generation and degradation is regulated by class III phosphatidylinositol-3-kinase (PI3K) complexes including Atg6/Beclin-1. Therefore, viruses often interfere with these complexes via targeting Beclin-1 in order to arrest autophagosome formation or maturation.

## 2. Beclin-1 Containing Complexes and Their Role in Macroautophagy

**Figure 1:**
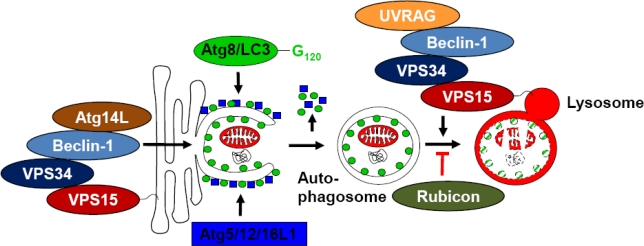
Atg6/Beclin-1 containing PI3 kinase complexes catalyze autophagosome generation and fusion with lysosomes. The Beclin-1 containing complex with Atg14L, VPS34 and VPS15 marks membranes at which the phagophore forms, which expands around its substrates with the help of the two ubiquitin-like molecules Atg8 and 12 and upon closure constitutes double-membrane surrounded autophagosomes. Their fusion with lysosomes is assisted by the UVRAG, VPS34 and VPS15 containing Beclin-1 complex. Recruitment of Rubicon into this complex blocks autophagosome fusion with lysosomes.

During autophagosome initiation Atg6/Beclin-1 is associated with the PI3K VPS34 and the adaptor proteins myristoylated VPS15 for membrane anchoring and Atg14L (Figure 1) [21–23]. This complex marks membranes for phagophore formation with PI3P, for example downstream of the starvation induced ULK complexes [24]. For incorporation into this complex Atg6/Beclin-1 has to dissociate from the anti-apoptotic Bcl-2 and Bcl-xL proteins, which anchor Atg6/Beclin-1 at the endoplasmic reticulum membrane. The Beclin-1 interacting protein AMBRA1 disrupts this interaction and thereby activates macroautophagy [25]. *Vice versa*, macroautophagy is compromised by mono-allelic deletion of Beclin-1 in 40–75% of ovarian and breast carcinomas [26,27]. These factors influence the generation of autophagosomes. In addition, the maturation, namely fusion of autophagosomes with lysosomes, is influenced by Beclin-1 containing VPS34 complexes. Instead of Atg14L, however, these contain the UV irradiation resistance-associated gene (UVRAG). This protein enhances autophagosome maturation, but again a negative regulator exists in the form of the Rubicon protein, which associates with the UVRAG containing Beclin-1 complex and arrests autophagosome maturation [21,22]. Thus, higher eukaryotic cells carry sophisticated molecular machineries for the regulation of PI3P deposition at future or formed autophagosomal membranes to react to metabolic needs, but also infections by regulating macroautophagy.

## 3. Macroautophagy in Pathogen Sensing

Pathogen sensing is the first step during the initiation of immune responses. Pathogen-associated molecular pattern (PAMP) receptors detect motifs in viruses, bacteria, fungi and parasites [28]. This recognition alarms the immune system by cytokine secretion and activation of antigen presenting cells. Different pattern recognition receptor (PRR) families monitor extracellular and intracellular space. RIG-I like (RLRs) and NOD like receptors (NLRs, including inflammasomes) detect cytosolic RNA and DNA, crystals or other danger molecules, respectively. In contrast, Toll-like (TLRs) and C-type lectin like receptors (CLRs) detect extracellular pathogen products. Interestingly, some TLRs signal from vesicular compartments and access to these endosomal TLRs (TLR3, 7, 8 and 9) can be regulated by vesicular transport. Along these lines it has been demonstrated that replication intermediates of Sendai and Vesicular Stomatitis virus require macroautophagy to be sensed by TLR7/8 [29]. Transport of these viral RNAs into TLR7/8 containing endosomes by autophagosomes was required for IFNα production by plasmacytoid dendritic cells (DCs) in response to infection. Similarly, macroautophagy has been shown to facilitate B cell receptor ligand transport to TLR9 containing endosomes for B cell activation [30]. Thus, PAMP sensing by vesicular TLRs of antigen presenting cells seems to benefit from macroautophagic transport of ligands to endosomes.

In contrast, cytosolic PRRs might be negatively regulated by macroautophagy. In the absence of Atg5, a component of the Atg8/LC3 E3-like ligase, dsRNA elicits increased IFNα production via RIG-I recognition in mouse embryonic fibroblasts [31]. Deficiency of macroautophagy leads to enhanced reactive oxygen species (ROS) production by dysfunctional mitochondria, which directly stimulates RLR activation [32]. ROS production does not only augment RLR, but also NLR signaling. Indeed Atg16L1 deficient macrophages produce elevated levels of inflammasome dependent IL-1 [33]. This elevated inflammasome activity is dependent on mitochondrial ROS, and inflammasomes and ROS contribute to mitochondrial DNA release into the cytosol, which augments IL-1 production [34,35]. Thus, PAMP recognition by vesicular TLRs of antigen presenting cells is supported by macroautophagy, while the same process dampens cytosolic PAMP recognition in somatic cells. This could benefit the initiation of immune responses by focusing activation towards antigen presenting cells, like DCs and B cells.

## 4. Contribution of Macroautophagy to Innate and Adaptive Immunity

During such immune responses macroautophagy restricts intracellular pathogens and allows for antigen presentation to the adaptive immune system. Indeed during the engulfment of group A *Streptococci* autophagosomes can reach ten-fold larger diameters (10 μm) than usually observed for metabolic autophagosomes (0.5–1 μm in diameter) [36]. Macroautophagy restricts these bacteria, which escape from endosomes to replicate in the cytosol. Interestingly, the formation of these large *Streptococci* containing autophagosomes requires Rab7, a GTPase that has previously been described to mediate autophagosome fusion to lysosomes [37]. In addition to bacteria, parasites like *Toxoplasma gondii* seems to be targeted by macroautophagy [38,39]. *Toxoplasma* conditions phagosomes to prevent their fusion with lysosomes, which allows the parasite to replicate in these vesicular compartments. The protective phagosome membrane, however, can be stripped by immunity related GTPase M (IRGM), which then exposes *Toxoplasma* to macroautophagy, leading to degradation in lysosomes [39]. Similar mechanisms might be at work during the restriction of *Mycobacterium tuberculosis* growth inhibition by macroautophagy [40,41]. Finally, neurotrophic Sindbis virus infection is also compromised by macroautophagy [42,43]. Enhancement of macroautophagy by Atg6/Beclin-1 overexpression [42] compromises Sindbis virus replication, while deficiency of Atg5 in neurons increases immunopathology of Sindbis virus infection in the central nervous system of mice [43]. While the detailed mechanism of viral restriction is not known so far, it might involve direct degradation of viral particles. In addition to simply using lysosomal hydrolysis for pathogen destruction, autophagosomes also deliver substrates to lysosomes, from which antibacterial peptides can be generated. These include ubiquitin [44] and the ubiquitin-domain containing ribosomal protein Fau [45]. Thus, macroautophagy can restrict bacteria, parasites and viruses during immune responses.

As a byproduct of this, pathogen degradation fragments are recycled for antigen presentation on MHC molecules to T cells. Especially, antigen processing for MHC class II presentation to CD4^+^ helper T cells utilizes lysosomal hydrolysis. Accordingly, intracellular antigen processing of some viral and bacterial antigens utilizes macroautophagy [46–49]. In good agreement with these examples of antigen processing by macroautophagy, autophagosomes have been found to frequently fuse with MHC class II loading compartments [50], and enhanced autophagosome formation due to starvation changes the repertoire of MHC class II presented peptides to contain more frequently ligands, which are derived from cytosolic and nuclear proteins [51]. This alteration of the MHC class II presented ligandome by macroautophagy leads also to deficient positive and negative T cell selection in the thymus [52]. Furthermore, even though mechanistically not very well understood so far, Atg5 deficiency in DCs compromises the development of CD4^+^ T cell responses upon herpes simplex virus (HSV) infection of mice [53], and loss of an HSV encoded macroautophagy inhibitor augments CD4^+^ T cell responses during infection [54]. These studies suggest that degradation of pathogens via macroautophagy leads to MHC class II presentation of their fragments for stimulation of CD4^+^ T cell responses.

## 5. Inhibition of Autophagosome Generation by Viral Proteins Interacting with Beclin-1

**Figure 2:**
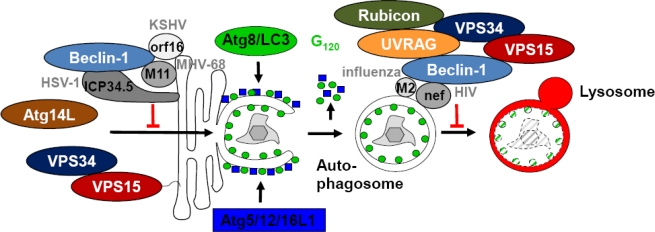
Viral proteins block autophagosome generation and degradation via Beclin-1 binding. KSHV orf16, MHV-68 M11 and HSV-1 ICP34.5 anchor Beclin-1 at membranes and block its incorporation into the PI3 kinase complex with VPS34, VPS15 and Atg14L, which catalyzes autophagosome generation. Furthermore influenza A virus M2 and HIV nef presumably stabilize through their Beclin-1 binding inhibitory PI3 kinase complexes that prevent fusion between autophagosomes and lysosomes.

However, viruses counteract these beneficial functions of macroautophagy for immune responses. They have developed strategies to both target autophagosome generation and autophagosome maturation. A rich source of inhibitors of autophagosome generation is the herpesvirus family (Figure 2 and Table 1) [55]. In fact, all three herpesvirus subfamilies encode inhibitors of macroautophagy, which primarily target the Atg14L containing Beclin-1 complex of VPS34 and VPS15. The α-herpesvirus HSV-1 encodes the ICP34.5 protein, which interacts with Beclin-1 via a 20 amino acid region (aa 68–87) and thereby compromises autophagosome generation [56]. HSV encoding ICP34.5, in which this region has been deleted, demonstrates attenuated neurovirulence in mice. In correlation with this attenuated neurovirulence, autophagosome accumulation can be observed in mutant virus infected neurons, and loss of the double-strand RNA-dependent kinase PKR, which stimulates macroautophagy in HSV infected cells, eliminates the protective function of elevated macroautophagy against mutant HSV. Without ICP34.5, HSV particles can be found in autophagosomes by electron microscopy [57,58]. In addition, loss of ICP34.5 also elevates HSV specific CD4^+^ T cell responses during infection [54]. Besides HSV, γ-herpesviruses also encode Beclin-1 interacting proteins. Kaposi sarcoma associated herpesvirus (KSHV) encodes a viral Bcl-2 homologue with its open reading frame (orf) 16. This binds Atg6/Beclin-1 with higher affinity than cellular Bcl-2 [59]. Binding of viral Bcl-2 inhibits macroautophagy by interrupting Beclin-1 incorporation in VPS34 complexes, probably via anchoring Beclin-1 at endoplasmic reticulum membranes [60]. Similarly, the viral Bcl-2 homologue M11 of the murine γ-herpesvirus 68 (MHV-68) interacts with Beclin-1 and inhibits autophagosome formation [61]. It binds with higher affinity to Beclin-1 than cellular Bcl-2 and Bcl-xL. Three amino acids at positions 85–87 are required for Beclin-1 binding by M11, and a C-terminally, but not N-terminally truncated molecule can still interact with Beclin-1 [62]. Interestingly, recombinant viruses lacking either the N-terminal α1 helix or with mutated amino acids 85–87 no longer inhibited macroautophagy upon infection, and loss of macroautophagy inhibition compromised persistence of MHV-68 *in vivo* [62]. Thus, herpesviruses sequester Beclin-1 to inhibit autophagosome formation to enhance their neurovirulence and increase their persistence *in vivo*.

## 6. Immune Escape from Autophagosome Maturation via Targeting of Beclin-1

In contrast to the DNA viruses of the herpesvirus family that inhibit autophagosome generation, RNA viruses seem to stabilize autophagosomes by preventing their degradation. Indeed, both infections with the hepatitis C virus and poliovirus accumulate autophagosomes, and benefit for their replication from this macroautophagy regulation [63–66]. However, for these and most other viral infections, this autophagosome accumulation is mechanistically not well understood. However, in two instances, influenza and human immunodeficiency virus (HIV) infection, some insights into mechanisms of autophagosome accumulation have been gained, and both pathogens target Beclin-1, presumably stabilizing complexes of Beclin-1, VPS34, VPS15, UVRAG and Rubicon that inhibit autophagosome maturation (Figure 2 and Table 1). Influenza A virus infection stabilizes autophagosomes, which fuse to large perinuclear vesicles and contain viral proteins, but not any lysosomal markers [67]. Matrix protein 2 (M2) of influenza A virus can induce this autophagosome accumulation, is localized to these accumulating autophagosomes, and M2 deficient virus does no longer establish this block of macroautophagy. Interestingly, the main function of M2, its proton channel activity that is required for viral core acidification during infection, does not seem to be involved in autophagosome stabilization. Instead full-length and a truncated M2 version (aa 1–60) are able to interact with Beclin-1. Inhibition of autophagosome maturation compromised survival of influenza A virus infected cells, thereby enhancing the pro-apoptotic effect of the viral protein PB-F1 by blocking the pro-survival pathway macroautophagy. Under certain infection conditions, this regulation of macroautophagy might also benefit viral replication [68]. Furthermore, consistent with inhibition of autophagosome maturation, influenza A virus antigens are not processed intracellularly for MHC class II presentation to CD4^+^ T cells [69]. Instead, proteasomal degradation facilitates intracellular influenza antigen processing for MHC class II presentation. This suggests that proteasomal degradation takes over when macroautophagy is blocked, and *vice versa* an increase in macroautophagy can be detected in cells treated with proteasomal inhibitors [66,70]. Interestingly, autophagosome generation facilitates antigen transfer from influenza A virus infected cells to neighboring DCs [71]. It is tempting to speculate that exocytosis of viral protein carrying autophagosomes, which accumulate due to the M2 inducted block of their fusion with lysosomes, might contribute to this cross-presentation. Similar to influenza A virus, HIV blocks autophagosome maturation in infected macrophages [72]. HIV gag proteins accumulate in the stabilized autophagosomes and this autophagosome accumulation enhances viral replication in macrophages. The virus achieves this autophagosome accumulation via its nef protein, which co-localizes with autophagosomes and binds to Beclin-1 via its DD motif (aa 174–175) and this motif is required for autophagosome accumulation by nef. This fusion block is also established in DCs upon HIV infection, and prevents the formation of so-called immunoamphisomes [73]. These studies suggest that HIV and influenza A virus block autophagosome maturation by encoding Beclin-1 interacting proteins.

## 7. Conclusions

Macroautophagy, a pathway that has been originally appreciated for its pro-survival function in nutrient recycling during starvation, has in recent years been recognized for its functions during immune responses. Both pathogen sensing by innate and adaptive immune compartment, as well as pathogen destruction through this pathway are supported by macroautophagy. Accordingly, successful viral pathogens have learned to interfere with this pathway for their own benefit. While herpesviruses inhibit autophagosome generation, many RNA viruses, including influenza virus and HIV, block autophagosome maturation and degradation, but seem to benefit from stabilization of these vesicles. Interestingly, both inhibition of autophagosome generation and degradation seems to be often achieved through viral protein binding to Beclin-1, probably regulating complexes of this essential macroautophagy protein that are either involved in phagophore generation or autophagosome fusion with lysosomes. How this is achieved by the different viral proteins remains to be seen. However, these viral modulators of macroautophagy already now give us tools to experimentally regulate this catabolic pathway, and interference with their function might hopefully suggest some therapeutic interventions against the encoding viruses in the future.

## Figures and Tables

**Table 1:** Viral proteins that interact with Atg6/Beclin-1.

**Virus**	**Protein**	**Function**	**Reference**
HSV-1	ICP34.5	blocks autophagosome generation	[54]
KSHV	orf16	blocks autophagosome generation	[58]
MHV-68	M11	blocks autophagosome generation	[59,60]
HIV	nef	blocks autophagosome maturation	[70]
Influenza A	M2	blocks autophagosome maturation	[65]
